# Sustainability Comes to Life. Nature-Based Adventure Tourism in Norway

**DOI:** 10.3389/fspor.2021.686459

**Published:** 2021-06-11

**Authors:** Axel Rosenberg, Pip M. Lynch, Aage Radmann

**Affiliations:** Department of Teacher Education and Outdoor Studies, Norwegian School of Sport Sciences, Oslo, Norway

**Keywords:** sustainability, adventure tourism, nature, environment, ethnography, dramaturgy, performance

## Abstract

This paper investigates how tourists and guides perform sustainability during adventure tourism trips in natural environments. The paper draws on empirical data from an ethnographic study of five different multi-day trips in Norway, each of which used skiing, hiking, or biking as the mode of travel. In our analysis, we focus on how the different actors understood, operationalized and practiced elements of sustainability in their everyday lives while on the trips. The paper applies a micro-sociological perspective to the nature-based adventure tourism scene where the interplay between tourists, guides, adventure activities and nature is understood as multiple dialectic performances co-produced by the different actors. Goffman's dramaturgical metaphors, and concepts of frames, appearance, and manner saturate recent research on tourism and nature guiding. This paper builds on the “performance turn” as a theoretical point of departure for understanding sustainability in nature-based adventure tourism experiences. In participant observations and post-trip interviews with Norwegian and international tourists and their guides, we found that sustainability performances were not a major aspect of the trips. We did find some performances of mainly “light” sustainability and, among them, elements of ambivalence and ambiguity. Our data indicate that some guides tread a fine line between enhancing and deepening tourists' experiences of nature and sustainability or negatively impacting the perceived enjoyment imperative of the trip. International tourists expressed deeper sustainability overall. We reflect on the relative explanatory strengths of Goffman's “frames” and interaction order, and Persson's “framing,” for understanding the interplay between guide and tourist sustainability performances and conclude with pointers for teasing out the complexities we identify.

## Introduction

Tourism is one of the world's fastest growing industries and in recent years. Norway has experienced a marked increase in domestic and international tourism (Ministry of Trade, 2017). Norway's international reputation for being “sustainable” and environmentally conscious (Ministry of Trade, 2017) arguably creates certain expectations of the country as a destination. This paper investigates how tourists and guides perform sustainability during adventure tourism trips in natural environments. This is not a study of sustainable tourism, but of sustainability as expressed – or not – in tourism experience.

### Background

Tourism research is often characterized as multi- and inter-disciplinary as well as a fragmented in its scope (Benckendorff and Zehrer, 2013). In this paper, we draw from the literatures of nature-based tourism and adventure tourism. Nature-based tourism is often believed to “influence tourists' environmentally friendly attitudes, knowledge, and ultimately their behavior” (Ardoin et al., 2015, p. 838), however, in their review of the research, these authors found that “few studies have empirically documented these outcomes, and those that do are inconsistent in the variables measured and the time frame analyzed” (p. 838). Previous research on nature-based adventure tourism has typically surveyed guides, tourists, or both (Pereira and Mykletun, 2012; Ardoin et al., 2015), but few have gone into the field looking for how concepts of sustainability can ‘come to life in various ways’ during a guided nature-based adventure tour.

Guides function as narrators, social organizers and instructors, and are central to transforming an ordinary tourist experience into an extraordinary or spectacular and unique experience (Hansen and Mossberg, 2017). The extent to which, and ways in which, guides influence tourist understandings, knowledge, and behaviors of sustainability has been the focus of some international research (Powell and Ham, 2008; Randall and Rollins, 2009; Weiler and Kim, 2011; Pereira and Mykletun, 2012), without conclusive results, and to date the Norwegian context has not been studied.

Some of the international research has paid attention to tour guides as potential agents of change (see Zillinger et al., 2012; Jonasson et al., 2013; Rokenes et al., 2015; Vold, 2015; Weiler and Black, 2015; Jonasson and Smith, 2017) and there is evidence of a growing research focus on “the relationship between face-to-face interpretation/tour guiding and sustainability” (Weiler and Black, 2015, p. 76), at least in wildlife tourism (see Zeppel and Muloin, 2008; Ballantyne et al., 2009).

Tourists' expectations about what they will experience on a tour arise partly from the information provided by tour companies (Collado et al., 2009; Skinner and Theodossopoulos, 2011). If tourist expectations are not met, the companies risk reputational damage and subsequent financial impacts (Collado et al., 2009), so it is in each company's interests to prescribe to at least some extent the activities of their guides. Tour guides, then, “may thus feel relatively powerless to make a difference in contributing to the sustainability of a particular activity, tour, business, community, industry or environment” (Weiler and Black, 2015 p. 73–74). Our study includes a focus on guides' understandings of sustainability on tour and how those understandings impact their performances of sustainability.

The contemporary Norwegian context provides further impetus for this study. According to the most recent government white paper on tourism, nature is “still the most important reason the tourists choose Norway as a tourist destination” (Ministry of Trade, 2017, p. 31). Experience tourism is the fastest growing tourism sector (Fredman and Haukeland, 2017; Ministry of Trade, 2017) and tourism businesses that are based on nature-, food- or culture experiences represent the core of the Norwegian tourism product (Fredman and Tyrväinen, 2010; Ministry of Trade, 2017). From the government's perspective, it is “authentic” and “meaningful” experiences that should be the basis for tourism value creation, as it is in such experiences that travel motivation and willingness to pay lie. How sustainability can or should be operationalized while tourists are experiencing authenticity and meaningfulness in nature, however, is not discussed (Ministry of Trade, 2017).

A relatively united Norwegian travel industry supported the principles of the white paper with a “roadmap” titled “Toward a sustainable tourism industry.” In it, ‘high yield – low impact” nature-based tourism takes center-stage (NHO, 2017, p. 5) with physically active nature and cultural experiences based on the Norwegian tradition of outdoor life (friluftsliv). Friluftsliv – translated as “free-air-life” – is a Scandinavian practice of spending time in nature. Norwegian friluftsliv, in particular, emphasizes traditional modes of travel such as cross-country ski touring, hiking and biking, and “low” technologies, such as camping or staying in simple cabins and cooking on open fires (Odden, 2008). Friluftsliv is considered to be an important part of the (imagined) Norwegian national identity (Witoszek, 1998; Pedersen Gurholt, 2008; Gurholt, 2014).

The road map stresses that with a stronger global focus on intact nature, climate change and environmental quality, an increasing number of tourists seek destinations offering cleanliness, healthiness, and effective protection of culture and nature. By 2050, when eight out of 10 people worldwide will live in cities, an increasing number of tourists will avoid destinations characterized by hustle and bustle, noise and litter, and instead choose a journey that promotes the environment (NHO, 2017, p. 8).

Given the focus on sustainability, broadly interpreted, in both documents, we argue that it is relevant and timely to investigate what is going on in terms of sustainability at the micro-level of tourism experience in Norway. It could be argued that when guides choose to work in nature-based adventure tourism and when tourists choose to purchase a nature-based adventure tour, they are already performing sustainability, however that is not our focus in this paper. Our focus is entirely on what happens once the tour begins through to when it ends. We investigate the experiences of guides and tourists of an industry-leading Norwegian tour-operator for the purpose of discovering how they understand, operationalize and practice elements of sustainability in their everyday lives while on nature-based tours in Norway.

#### Sustainability

Much of the literature on nature-based adventure tourism, nature-based tourism and ecotourism refers to sustainability without providing an operational definition of it. We consider that contemporary conceptions of sustainability will enhance the reliability of our study and so we adopt Force et al. (2018) distinction between sustainable tourism and tourism sustainability. According to these authors, the former concerns the socioeconomics of tourism, especially at the local level. This is the main focus of the United Nation's Sustainable Development Goals for sustainable tourism. Member nations are expected to foster tourism in ways that create jobs, support local culture and new product development as well as in ways that protect environment values such as biodiversity, ecosystem health and more (United Nations (n.d.)). Tourism sustainability, in contrast, concerns “the design of tourism activities in ways that contribute to sustainability transitions globally” (p. 431). Our focus is on tourism sustainability. Sustainability transitions are “personal change[s] in tourists' identities” that lead to such things as active “commitment to environmental and cultural protection … nature-relatedness … [and tourists'] awareness of their relationship to the global collective” (p. 433). Our understanding of the term sustainability is also informed by Salas-Zapata and Ortiz-Muñoz' (2019) clarification of its use by researchers. We adopt the meaning “[s]ustainability as a set of guiding criteria for human action” rather than “sustainability as a goal of humankind” (p. 155), “sustainability as an object,” or “[s]ustainability as an approach of study” (p. 157). Criteria for guiding human action include, but are not limited to, such things as utilizing renewable resources, enhancing human well-being, avoiding ecosystem degradation, and generating social and cultural benefits. In this article, then, sustainability means a set of guiding criteria for personal change in tourists and guides toward deeper nature-relatedness, more active environmental and cultural protection, and stronger positive relationships to the global collective.

Nature-relatedness is defined as a degree of “connectedness to the natural world” and “comprises the cognitive, affective, and physical connection we have with nature” (Nisbet, 2021). Nisbet et al's (2009) nature-relatedness scale considers deep nature-relatedness to be expressed as a lot of time spent in natural spaces, preference for isolation in wilderness, self-identification as part of nature, awareness of environmental issues, and lifestyle changes in response to knowledge of, or feelings toward, nature. A light nature-relatedness is the opposite of these factors. Thus, sustainability might be expressed by nature-based adventure tour guides and tourists in one or more of the ways described on a continuum.

The Organization for Economic Co-operation and Development (OECD) defines environmental protection in terms of maintaining or restoring the quality of an environment (OECD, 2003). Environmental protection actions could include cleaning plastic pollution from rivers and lakes, protecting populations of threatened species, or donating money to environmental causes, among many other things. Cultural protection refers to protecting the material resources of cultural groups (Durie, 2008) such as artifacts, structures, monuments, language, intellectual knowledge and “places associated with historical events, beliefs, and traditions” (Cultural Heritage Act, 1978, § 2). Deep sustainability performances during nature-based adventure tourism trips might include much active interest in, or active participation in, these types of environmental and cultural protection. Light sustainability might include a few, or incidental, expressions of interest in these things.

Finally, a positive relationship to the global collective refers to attitudes of support for worldwide action on shared international problems such as climate change, large-scale pollution, disease, international aid, terrorism, and biodiversity loss (Sandler, 2010). Guides and tourists on nature-based adventure tourism trips might express strong positive relationships as part of their performances of sustainability. Others might express weak positive, or even negative, relationships as part of their light sustainability performances.

We used the concepts of nature-relatedness, action toward environmental and cultural protection, and positive relationships toward the global collective as guides for understanding the types of sustainability found in our data. In the Methods section, we describe how being “guided” by the concepts differs from being “driven” by them. Next, we define our study in relation to the existing literature on sustainability in nature-based tourism.

#### Nature-Based Adventure Tourism

Nature-based tourism, as a socio-cultural phenomenon (Sandell, 2003), has been defined in many, sometimes overlapping ways (Fredman et al., 2009, 2014; Fredman and Tyrväinen, 2010), such as adventure tourism, environmental tourism, ecotourism, and ecological tourism. At its most basic, nature-based tourism is related to places and objects that are not human-made, and visits and activities that occur beyond a person's familiar environments (Fredman et al., 2009). Hence, we adopt the widely accepted Scandinavian definition of nature-based tourism: “human activities occurring when visiting in nature areas outside the person's ordinary neighborhood” (Fredman et al., 2009, p. 24–25).

Our focus is on nature-based adventure tourism (Buckley, 2006, 2010; Mihalic, 2006; Rokenes et al., 2015; Beams et al., 2019) to foreground the sustainability aspects of commercialized nature tourist experiences that “often involve[e] perceived risk or controlled danger associated with personal challenges” (Mihalic, 2006, p. 114). Adventure tourism and nature-based tourism are closely related with some overlap in practice. However, “whilst nature-based tourism products focus on seeing … adventure tourism products focus on doing” (Buckley, 2010, p. 4). Thus, nature-based adventure tourism can be considered tourism products in nature that focus on both seeing and doing. In the Norwegian context, adventure tourism experiences commonly center on hiking and biking journeys in nature, skiing through forest or mountain environments, sea-kayaking, and mountaineering. What counts as “perceived risk,” “controlled danger,” and “personal challenges” is highly individualistic, however, “[f]rom the perspective of the individual tourist, anything which they personally consider adventurous can be counted as adventure tourism” (Buckley, 2010, p. 7). For our purposes, we accept the types of physical activities mentioned above, when conducted in guided tours in natural environments, to constitute nature-based adventure tourism.

### “What Are We Doing”

Experiences of sustainability in tourism are, arguably, important for several reasons of which the most pertinent to this study is that tourism experiences can have educational effects which can contribute to wider public understandings and motivations toward sustainability (Ballantyne et al., 2010; Force et al., 2018; Winter et al., 2020). Understanding “what is it that's going on” (Goffman, 1974, p. 8) regarding sustainability in nature-based adventure tourism allows researchers, policymakers, tourism operators, guides and tourists to respond in ways that further their respective ambitions of sustainability at national, industry, professional, and personal levels, respectively. We take a Goffmanian approach to investigating if and how different actors – the tourists and the guides – understand, operationalize, practice and embody nature-relatedness, active environmentally friendly behavior, and positive relationships to the global collective. As we next explain, taking an ethnographic approach allowed us to focus directly on “performances” of sustainability, a novel approach to the topic in nature-based adventure tourism.

### Theoretical Framework

The “performance turn” (Edensor, 1998, 2000, 2001; Haldrup and Larsen, 2010; Larsen, 2010; Urry and Larsen, 2011; Larsen and Meged, 2013) in tourism research, however, and despite some criticism (Saldaña, 2006), has re-imagined the guided tour as “created by a relational praxis that builds on and involves bodily and verbal negotiations, fluid power relations and interactions between tourists and guides and between tourists” (Larsen and Meged, 2013, p. 100). It can be traced back to new ways of investigating, analyzing and understanding tourism, starting in the late 1990s (Edensor, 1998, 2001; Larsen, 2010; Urry and Larsen, 2011; Cohen and Cohen, 2012; Jonasson and Scherle, 2012; Larsen and Meged, 2013). Although performances can be considered to be, in part, *pre*formed, they are not absolutely fixed. The performance turn emphasizes “creativity, detours and productive practices” (Larsen and Meged, 2013, p. 89), and “relates to the theatrical perspective and invokes **enactment** by performers or actors of a role or scripts, as well as display for an audience. Performances involve pretense” (Harwood and El-Manstrly, 2012, p. 15, bold in original). More recent research on guided tours has shown how tourists contribute to the co-creation of guided tours both alongside the guide, as well as in opposing and contradictory ways. Larsen (2010) and Urry and Larsen (2011) claim that the performance turn has “challenged representational and textual readings of tourism … by making “ethnographies” of what humans and institutions do – enact and stage – in order to make tourism and performances happen” (Larsen, 2010, p. 323). Consequently, the performance turn represents a move to ethnographic research in tourism. The aim of ethnographic approaches is to “go beyond the abstract models and frameworks of attitude-behavior connection …[and] to explore in greater detail how practices are performed and negotiated *in situ*” (Hargreaves, 2016, p. 57).

According to Vold (2015), nature guides choose which aspects of nature to focus on and by doing so they greatly influence how tourists understand and experience nature and tourism. However, nature-based tourism guides might also be constrained in their choices of focus because they are employed by tour companies that have certain obligations to their clientele (Prakash et al., 2011).

In this paper, we investigate tourists' and guides' understandings and experiences of sustainability in nature-based adventure tourism through their performances. This work contributes to a new perspective to understanding sustainability in tourism, and especially in face-to-face relations in “real (tourism) life.” Recent tourism research has drawn on Goffmanian concepts (Edensor, 1998, 2000, 2001; Larsen, 2010; Urry and Larsen, 2011; Jonasson and Scherle, 2012; Larsen and Meged, 2013; Williams, 2013) to understand the face-to-face interactions between tourists and between tourists and guides. The idea that tourists and guides manage the impressions they make on others in social situations emanates from Goffman's (1959) theory of social interaction, in particular the ideas of “frontstage” and “backstage” performances, frames, lines, face, and the interaction order. In all social situations, Goffman (1959) argues, people want to present themselves so that the “audience” perceives them to be as they wish to be perceived. Performances are designed to make a particular impression on the other people present through “patterns of verbal and non-verbal acts” that Goffman (1967, p. 5) called “lines.” The “frontstage” concerns how people present themselves within the immediate social surroundings and how they are perceived by others in the same immediate environment.

Self-presentation, or “face” may be defined as “the positive social value a person effectively claims for himself by the line others assume he has taken during a particular contact” (Goffman, 1967, p. 5). The “face” adopted by any one person depends on who the “audience” is and what the situational norms are (Goffman, 1959; Jacobsen and Kristiansen, 2015). In the “backstage,” people relax and take off their “face-masks” of social performance (Goffman, 1959; Jacobsen and Kristiansen, 2015). From this perspective, guided tours can be viewed as dialectical, as shaped by the interplay of performances by the guides and the guided (Urry and Larsen, 2011).

The interplay of “lines” and performances operates through individual “frames” (Goffman, 1974). Frames are operable within social situations, or “social frameworks” in Goffman's (1974) typology. As Persson (2019, p. 49) explains, Goffman saw social life as social situations shared by individuals, none of whom have “fully reliable knowledge” about one another and so each individual must interact with others at the same time as seeking information about how best to interact. Individuals therefore need to quickly define the situation they are in and this definition is what Goffman called a “frame.” A frame is an “organization of experience” (Goffman, 1974, p. 11) and “a different scheme of interpretation for the meaning of an act” (Goffman, 1974, p. 231). This concept of *frames* “emphasized its simultaneously cognitive, social interactive, and situational aspects” (Persson, 2019, p. 49). By asking Goffman's question – “what is it that's going on here?” – it becomes apparent that the answer needs to be “seen in the light of its context” (Persson, 2019, p. 49) and so also asks the question of “what [social rule or norm] applies here?” Persson (2019, p. 65).

Goffman's (1959, 1967, 1974, 1983) research centers on what he termed the “interaction order” and the “expressive order” both of which are essential for understanding social interaction. Our collective understanding of these terms is that they are closely related but distinguished by scale. At a larger scale of social interaction, the interaction order aligns roughly with social norms but with a focus on interpersonal interaction rather than social structures or power. It is the shared understandings individuals have of acceptable behavior in particular settings, allowing them to respond to the questions “what is it OK to do here?” and “what possibilities for behavior does this setting open for me?.” Examples of behaviors in the interaction order include maintaining culturally appropriate personal space, keeping right (or left) on footpaths, sitting and quietly watching a movie in a movie theater, dancing and singing aloud in the arena of a rock concert. In these examples, individuals are in face-to-face contact but not necessarily directly interacting with one another. Our collective understanding of the expressive order, on the other hand, aligns more with manners, or the smaller scale, more detailed level of social interactions. These include the shared understandings of acceptable verbal and non-verbal communication between persons in direct face-to-face situations. The “expressive order” is “an order that regulates the flow of events, large or small, so that anything that appears to be expressed by [a person] will be consistent with his face (sic)” (Goffman, 1967, p. 9). As we understand it, the expressive order allows individuals to respond to the questions “how is it OK to respond to the other person/s here?” and “what possibilities for response are open to me here?.” An example of the expressive order related to our research topic would be tourists paying attention when guide is explaining the how to prepare for the day ahead (e.g., by facing the guide, making eye contact if culturally appropriate, acknowledging them by uttering “mm” or nodding one's head).

Finally, and importantly, Goffman theorized that if someone challenges or breaches the interaction order or the expressive order, intentionally or not, a corrective process begins to either re-establish the original order or negotiate a new order from the “cognitive presuppositions” shared with the others in the setting (Goffman, 1983, p. 5). The corrective will be one or more “face-saving” practices (Goffman, 1967).

In this paper, we interpret nature-based adventure tourism as a social framework within which guides and tourists understand and respond to the interaction order and the expressive order during their encounters with one another. We approached the empirical study from a theoretical viewpoint that an individual's “cognitive presuppositions” shape their “frame” and inform their performances of sustainability while on nature-based adventure tourism trips. We continue by describing our applied methodology and our research and analytical methods, before reporting our findings.

## Methodology

For this study an ethnographic approach was deemed appropriate because it “allows one to gain information on tourist action and the embodied, tacit dimensions of nature-based tourism” (Rantala, 2011, p. 151) and “simultaneously allow[s] the observation of social and situated practices and participation in them” (Rantala, 2011, p. 153). An ethnographic approach is appropriate when the aim is to capture the micro-sociology, the information “given” and “given off” (Goffman, 1959; Rantala, 2011; Persson, 2019), the embodied as well as tacit practices, and the multitude of different performances that are enacted in and through social situations in nature-based adventure tourism. Our ethnographic fieldwork paid attention to how people talked, words and phrases they used, how they interacted with each other and with the environments they traveled through, where they gazed, how they embodied the landscape, what the guides emphasized or not. Rather than look for specific pre-determined verbal or non-verbal expressions, our aim was to remain open to whatever practices occurred in the field and then consider them in light of the concepts of sustainability discussed above and in the light of the national and industry sustainability focus.

In order to find out “what is it that's going on here,” we focused on tourist participants, tour guides, and the interactions between them. To do this we drew data from multiple, diverse trips offered by a nation-wide, industry-leading tourism operator. In the absence of an agreed definition of what constitutes “industry leading,” we selected one of the oldest nature-based adventure tour operators in Norway that has one of the most extensive tour catalogs. The selected operator offers trips throughout and beyond Norway and has been involved in sustainability discussions at a national level and in sustainability projects internationally. However, their website and brochures (checked during research design phase fall 2017 and immediately pre-fieldwork summer 2018) show that they do not actively market their trips as having a sustainable focus or credentials. Further, this operator could provide the best opportunities for participant observation, including as an apprentice-guide-researcher.

In this embedded single-case design (Yin, 2014, p. 50), guides and tourists make up the different embedded units of analysis and “the circumstances and conditions of an everyday situation” (p. 52) are those that occur on the guided nature-based adventure tours. Our decision to select a single tour company was informed by Flyvbjerg's (2001, p. 77) conception of “critical cases” for enhancing validity. Critical cases are those that are “either “most likely” or “least likely” … to confirm or irrefutably to falsify propositions and hypotheses” (Flyvbjerg, 2001, p. 78). An “extreme” critical case, such as the industry-leading tour operator in this study, enabled us to “achieve the greatest possible amount of information” (Flyvbjerg, 2001, p. 77) on our topic, which a representative case or random selection cannot do with as much certainty.

Five different tours make up the ethnographic material. All the tours took place in Norway between summer of 2017 and spring of 2018, and in different geographical locations: one in a mountainous part of central Norway (A); one along the coast of northern Norway (B); and three in the arctic high-mountain plateau of the northernmost part of Norway (C–E). Tour A took place late summer with only international tourists. Tour B took place early autumn, also with international tourists. Tours C, D, and E took place in the winter months with mainly Norwegian and some other Scandinavian tourists (from Denmark, Sweden, and Iceland). The tours varied in duration. Tour A, C, D, and E were 4 days each, while tour B spanned 8 days. In total 24 days were spent in the field. A total of 62 tourists and six guides were part of the study.

The study was approved by and conducted according to, the ethical guidelines of the Norwegian Center for Research Data (NSD) and The National Committee for Research Ethics in the Social Sciences and the Humanities (NESH). All participation in the study was voluntary, on the basis of anonymity, with the option of withdrawing at any time up to acceptance for publication. Participants were informed prior to, and written consent to observe all aspects of the trips, including social chats, and for post-trip interviews was obtained on the 1st day of each trip. Data was gathered through participant observation and interviews, as detailed below.

A general rule of participatory observation is that the researcher participates in the social interactions of the research context while at the same time striving not to influence those interactions significantly (Fangen, 2010, p. 80; Zahle, 2012, p. 54). However, participant observers cannot totally decide their field roles in advance. Roles and the degree of participation are usually in continuous (re)negotiation throughout the fieldwork (Spradley, 1980; Fangen, 2010; Wadel, 2014). Importantly, Wadel (2014) points out that roles open and close for different possibilities and associated data, and thus recommends that participant observers take on different roles so that they can study the field from a variety of perspectives.

Throughout the five tours in this study, the first author utilized various degrees of participation, involvement and observation to gather data, primarily participant observer and partially participant observer (Bryman, 2016, pp. 433–436; see also Spradley, 1980; Fangen, 2010; Wadel, 2014). In addition, on trips C and D, he was an apprentice-guide. This role gave him affordable access to the trip and the benefit of closeness to the guides' perspectives. It also provided “backstage” access to tourists' “backstage” spaces that would have been inappropriate otherwise. One of the guides' responsibilities on these trips was to check on each participant each evening to find out how well they were coping with the physical and other demands of the trip. Often, guides would be invited in to the tourists' accommodation (or invite themselves) and engage in social chat or be questioned about aspects of the trips. In this way, the field researcher gained additional access to tourists' “frames.” While working as an apprentice-guide, the first author aligned his professional frame with the lead-guide's apparent frame and reflected on this alignment in the reflective journal. The first author's opportunity to take on this dual role as both apprentice-guide and researcher gave him valuable first-hand experiences and helped deepen his understanding of the field.

The first author can be considered an insider in the field of nature-based adventure tourism through both his educational and work background. To obtain and maintain analytical distance (Spradley, 1980; Fangen, 2010) in the various roles adopted in the field, the researcher kept a reflective journal (Spradley, 1980; Saldaña, 2016) and used a field diary and voice recorder for field observations. He wrote the reflective journal throughout the fieldwork phase in order to become aware of any preconceptions and to increase introspectiveness (Spradley, 1980). Detailed observations were recorded throughout each day and were assisted by pre-prepared descriptive questions, such as “how do tourists talk about themselves, nature, and their experiences?” “what do the guides focus on/give emphasis?,” “how do tourists behave while on tour?,” “how do guides behave while on tour?.” These questions were also condensed into laminated, pocket-sized field cards that helped the researcher stay on-task throughout the fieldwork.

Twenty-nine participants and five guides were interviewed by the field researcher between 3 and 12-months post-trip (mid 2018 to mid 2019), using a semi-structured interview guide. The average duration of interviews was 1 h and 15 min, and the interview questions began very broadly (e.g., “tell me about the trip”) and became more focused as the interview progressed. If the interviewees had not mentioned sustainability themselves, the topic was brought up by the interviewer late in the interview. Twelve of the post-trip interviews were done face-to-face, while the majority, for logistical reasons, were conducted by digital videoconference or phone. The limitations of physical distance to qualitative interviewing (Bryman, 2016) were arguably offset by the fact that rapport had already been established between the interviewee and the interviewed, as they had spent many days living closely together while on tour.

### Analytical Approach

Interviews were transcribed verbatim using the Computer-Assisted Qualitative Data-Analysis Software (CAQDAS) MAXQDA. We used AI-transcription software with manual checking to transcribe eight interviews. All fieldnotes were transcribed and imported to MAXQDA. MAXQDA was used to code interview transcripts and fieldnotes. The use of CAQDAS has been criticized by some for influencing and enforcing a specific method to the analytical process (Kuckartz and Rädiker, 2019). However, we used CAQDAS as a “method-neutral toolbox” (Kuckartz and Rädiker, 2019, p. 9) that aided data organization for analysis (Ribbs, 2014).

The first author performed all interviews, transcribed all interviews and fieldnotes, and coded the transcribed material. All interviews were conducted in English or Norwegian, as the interviewee preferred. All authors are fluent in English; the first and third author are native speakers of Norwegian and the second author has a working knowledge of the language. The first author coded the data in both Norwegian and English and manually translated the excerpts quoted in this paper. To avoid known pitfalls of solo-coding (Saldaña, 2016; Braun and Clarke, 2019) and to strengthen coding validity, any coding uncertainties were discussed with the second and third author. The second and third author also read some of the interviews. The coding process started during the process of transcription with “preliminary jottings” (Saldaña, 2016, p. 21) and continued with an initially inductive, data driven, coding approach, through which themes were generated. Braun and Clarke (2019, p. 592) define themes as “stories about particular patterns of shared meaning across the dataset” and “underpinned by a central organizing concept” (p. 589). For this study the “central organizing concept” was that of “sustainability performances.” Once themes were generated, the data corpus (Braun and Clarke, 2006) was read iteratively with definitions of sustainability. In this way, Force et al. (2018) distinction between sustainable tourism and tourism sustainability, and Salas-Zapata and Ortiz-Muñoz (2019) clarification of sustainability as “a set of guiding criteria for human action” (p. 155) guided rather than drove the analysis, in that they became an analytical framework for organizing the different performances of sustainability identified in the data analysis. In this sense the analytical process could be considered that of a combination of “inductive” and “theoretical thematic analysis” (Braun and Clarke, 2006, p. 83–84).

## Results – “What is it That's Going on Here?”

In our data, we identified 11 types of sustainability performances. These are: noticing nature, desiring isolation in nature, responding to global issues, reducing pollution, supporting others' sustainability performances, minimizing environmental degradation, reflection on human/nature, connecting with nature, modeling sustainability performance, choosing tour operator, and learning about nature and culture. We also found performances not related to sustainability. While at first these results seem clear cut, they point to ambivalence and ambiguity in guides' and tourists' performances of sustainability in nature-based adventure tourism. We identify as ambivalence the low level of deliberate focus on sustainability during the trips generally and apparent randomness with which it occurs when it does. The ambiguities are one challenge and one conflict. The challenge is between sustainability performance and enjoyment, and the conflict is between sustainability performance and logistics. These are all detailed below and subsequently discussed in relation to the claims and criticisms of performativity and frames (see also Table 1).

**Table 1:** Sustainability themes/performances.

**Sustainability performances**	**Representative quotes from tourists**	**Representative quotes from guides**
Noticing nature	“The views, oh, I mean, hiking on a glacier and the beautiful … the blue of the ravines, you know, the crevasses and everything. Oh yeah. The vistas were amazing.” (International Tourist#7, trip 1) “We're just totally immersed in it … So I don't know, you see it. You smell it. You feel it. You don't just look at it. In a car you just look at it. The way we did it is totally immersed in it. So, it's like the difference between in a car. It's like looking at an aquarium and the way we did it. It's like jumping in the ocean. It's … everywhere around you, it's part of you, and in you. Both the good and the bad weather and everything else you feel and smell and hear, you really feel like you're part of it … I really felt how … I don't know. I have a different feel for nature and the landscape. And, you know, it doesn't, it just doesn't feel as isolated when you're like that with it.” (International Tourist#10, trip 1) “While on tour, I've become better at using my camera, 'oh, I must take a picture of this, and this, and this'.” (Norwegian Tourist#10, trip 4)^*^	“It is, kind of, many of the reasons for why people fall behind the group is (1) they might not be physically fit, but also because they take an extreme amount of pictures, and want to absorb what they see.”(Guide#1, trip 1)^*^ “It is mostly on the [bike trip] where we have tried to think about getting an early start so that we do not have too much, because it is busy there, you know, that we get moving before there's a lot of other people there, passing us, or, yes.” (Guide#1, trip 1)^*^
Desiring isolation in nature	“It's much more pleasurable to not be where there's a ton of people. I don't mind seeing a few people on the trail or seeing others, but if it's crowded it just takes away the experience, if it's too crowded.” (International Tourist#7, trip 1) “I feel in a way that my thoughts aren't as accessible, if I keep meeting other people all the time, aren't able to relax as much outside … I sort of feel that nature is so much stronger when there aren't … that many people around or other types of, eh, sort of social impressions, kind of.” (Norwegian Tourist#5, trip 3)^*^ “A lot of people, is disturbing, or sort of, it becomes overwhelming. You know, I'm there seeking nature, first of all. Not other people.” (Norwegian Tourist#15, Trip 4)^*^	“It is, there are some that sort of, maybe react on, so, if there are too many people there…It appears that people are more concerned with. That it might ruin the nature experience, or the experience as a whole.”(Guide#1, trip 3)^*^
Reducing pollution	“No, I cannot remember that it [sustainability] was a theme at all … No, I didn't perceive that it was a theme, neither among the participants nor from the guides.” (Norwegian Tourist#5, trip 5)^*^ “The … guides. emphasized and, and they, that we needed to, you know, bring back whatever we've take, you know … You know, we're going to bring back everything that we. even the trash and stuff like, we don't leave it there.” (International Tourist#3, trip 1)	“Trash. I'm very concerned with that. And I feel that everyone is very respectful of…that. Because, and it is, and [I] take out the doggie-bags, you know, actual doggie-bags to put toilet paper in. Nobody(!) says anything. Not a bad word. So, there I'm very straight forward. This is our toilet, but you won't leave anything behind. And that is totally okay.” (Guide#1, trip 4)^*^
Modeling sustainability performances	“I know we have a good talk at, even at the very beginning, about to leave no trace principles and that we were going to follow that. And then again, even things as simple as taking, the guides always had trash bags so that, you know, there wasn't going to be a problem of being able to clean up after ourselves and take care of what we need to take care of.” (International Tourist#10, trip 1) “And then it was this here that we should not leave anything behind us … that it should not seem that we had been there … And … this with, yes, [the guides] didn't state directly that we should not litter, but we understood that with the doggie-bag when we … if we were going to the bathroom, to put paper in, and so on.” (Norwegian Tourist#7, trip 4)^*^	“Yes, it's a bit about just showing that you pick up trash without saying anything. I do not need to talk about trash. I can just pick it up … and then I experience sometimes, then they start to pick themselves.”(Guide#2, trip 1)^*^ “I basically think that … being on a trip, in principle, is maybe … well, one of the most important things about it, because then they see … see the surroundings and the possible effect on … nature, where one is. But especially to be outside and fall in love with it [nature], like [skiing up north] and you've never been there before and then seeing it, then you might become more aware, or you see plastic in the ocean … it becomes a bit more real then. And I think that is an important part of it too.”(Guide#1, trip 3)^*^
Thinking about sustainability	“I don't think it [sustainability] was a topic of discussion as much as just clearly understood.” (International Tourist#11, trip 1)	“I experience a bit, you know, that people go on holiday to [laughs lightly] forget unpleasant things … So, I do not know how much, how much focus on … if it [sustainability] should somehow have a bigger part, that is sort of, not just a natural part that one talks about, but a thing that you are in a way forced upon. I'm, yes, a little unsure about that.” (Guide#1, trip 3)^*^ “It sort of, yes, some really do not care at all, and some are, very concerned about it [sustainability]. And I think, I also think a lot of people think like “Yeah, but” [laughs], if you know what I mean? And it's maybe most of them.” (Guide#2, trip 5)^*^
Supporting others' sustainability performances	“Weren't we very good at taking everything with us? At least I saw that people ran after and “oh-oh-oh-lost something”, and. So, it seemed as if they were concerned that there should be nothing left behind.” (Norwegian Tourist#10, trip 4)^*^	
Minimizing environmental degradation	“I sort of remember some things that were said about the way in which we would do, you know, we would walk or, do certain things … that would make it … that we would be careful on any path we were on … I sort of remember you know, being steered toward staying in one particular area and not, not treading on plants that if we stepped on would be, you know, take many years to recover.” (International Tourist#6, trip 1)	“Most people are very concerned about it, and if very many ask like, can we go off the beaten path? Will it be damaged then? And I generally think, by and large, a lot of respect for, and that goes for both international and Norwegians, but Norwegians do not ask that many questions, because they're used to it.”(Guide#2, trip 1)^*^
Reflection on human/nature	“That's exactly what I like to do. I think it's important to sit … and just absorb it, because we rush around so much … The thing I love about that outdoor-stuff, is eat, sleep, exercise and move on. You know, I spend most of my day thinking or … being mentally active. I feel I find, I need that balance of exercise, being outside, that connection, because it's … I don't get it in my work-life. And so … It is the quintessential stopping to smell the roses moment” (International Tourist#4, trip 2) “I like it … because I, I mean, I do yoga, where we're left to sort of meditate occasionally, so, so, and reflect. So that's quite, quite nice. And particularly if you're in a new scene, you know, in a new country, a new place … you can take in the environment and just think. Yes, it's really good.” (International Tourist#6, trip 2)	
Choosing tour operator	“… an agency … that shows that they've got good values in terms of the environment. And you can sense that quite often with the advertising and even speaking to the people. I often found out prior to booking, I'll have a chat with the staff … And I also, often the advertising says that they are environmentally sensitive from the point of view of a litter, take litter home. You know, they work on certain procedures in terms of being careful where to walk and be sensitive to the locals. So, a lot of it is, it comes through the text as well. It's reading between the lines, I think.” (International Tourist#6 Trip 2) “I cannot say that it … well, it's a little difficult to calculate if [the tour operator], if they do it in a way like this. But I think that's an important part, definitely. Absolutely. But I cannot say that it was, it was not a reason or, it was not like I felt that, they do it this way, and therefore I choose them.” (Norwegian Tourist#12, trip 3)^*^	
Learning about nature and culture	“I wanted to be able to … extricate myself from the group sometimes, just to be able to learn more about Norway and its people. So the easiest way to do that was to talk to our guides a lot … So my expectations for myself was to be very intentional about learning about Norway, the people, culture.” (International Tourist#9, trip 1) “But it's interesting when you travel because I'm looking at landscapes and I'm looking at, you know, birds and wildlife … I'm interested in the wildlife … I like meeting people and seeing how people live and see the differences in how people live, and also see places and, you know, hiking and getting into nature shows you things that, you know, are quite unique to an area. Sometimes it's fauna and sometimes it's flora or sometimes it's just landscape.” (International Tourist#4, trip 2) “It's kind of the experience that is … mostly for me, should I be completely honest. Yes, just being there … That is more important to me, kind of, than to stand looking out over the landscape … For me it is more important to do that trip, rather than to go and look at the surroundings, really.” (Norwegian Tourist#7, trip 5)^*^	“And I also think, foreigners then, for example, are often very attentive to the other, the new, or the other that they visit. So it is, they are not only interested in Norwegian nature, but they also appreciate being involved in Norwegian myths, folklore and history. And I think it also enriches their experience of being here, to a large part.” (Guide#2, trip 5)^*^
Responding to global issues	“I mean, it's … it's difficult, particularly if you want to … visit places, particularly, you know, outside your own country. I mean, you're adding to the carbon footprint by flying there (laughs).” (International Tourist#11, trip1) “I think, well, yes, a bit, not much, but I've become more and more aware, what sort of choices I make … And how I behave. That I should not disturb nature when it is at its most vulnerable.” (Norwegian Tourist#7, trip 4)^*^ “It's not sustainable that I should drive hundreds of miles up and … just to go skiing, it's not really, you know … But it's fantastically delightful … But sustainable? [laughs].” (Norwegian Tourist#10, trip 3)^*^	“Yes, I think they are interested in it … And that, yes, they wonder a bit how we do things, things here, in terms of energy and, yes … but they have still chosen to travel, they have chosen to travel far.” (Guide#1, trip 1)^*^ “When we're out on that glacier and it's melting away. Then there is quite a lot of focus on how far, how far it has retreated, how much it retreats during a year. How much do you notice global warming here? Those are questions I get, pretty much every time.” (Guide#2, trip 1)^*^ “It seems that very many of the guests I have had are relatively aware of it, especially flying, and, and things like that, and have brought up things by themselves, and of course, have a bad conscience for it, but do it anyway.” (Guide#1, trip 3)^*^

* *Quotes translated from Norwegian to English*.

### Noticing Nature

Throughout each trip the tourists noticed and regularly commented on the scenery, the wildlife, the vistas, the local culture, the “lack of other people,” the quietness, the fresh air, the experience of journeying through a landscape. Nature took center stage regardless of the travel mode in the different tours. Photography was another dimension of noticing nature. The tourists photographed the landscapes they traveled through, elements of those landscapes, and nature, and themselves or others in nature. Both international and Norwegian tourists stated in their interviews that the act of taking photos, and sometimes even *thinking* about taking photos, made them notice nature more.

### Desiring Isolation in Nature

Many of the Norwegian participants enjoyed being given time and place to just be “alone” together outside, to think about everything and nothing, to listen to their own breathing, find their own rhythm, feel and listen to the wind. Our summary of the tourist's perspective is that they want to get what they paid for: the experiences (hard earned), vistas and the solitude in nature as promised by the images in the company's brochure. On field trip A, for example, as the group traveled from the high-mountain and down to the coast, they encountered more and more people along the way until they reached a small coastal town. For one of the participants this town “was overly crowded with tourists” which they later stated was quite a shock and a negative experience for them. One of the main reasons this international tourist had come to Norway and do this particular trip was because they expected few other people there and they were disappointed to have come from the solitude of the high mountains and suddenly find themselves in a crowded tourist trap.

### Reducing Pollution

When guides addressed concepts of sustainability it was related to “leave no trace” (https://lnt.org). How this topic was addressed varied from guide to guide. Some gave an introductory talk the first day, emphasizing that if a person needed to use a toilet while out hiking, biking, or skiing, they should do so but dispose of the toilet paper in the doggy-bags made available by the guides. All but one of the guides highlighted the importance of not leaving any trash behind, using the doggy-bags for one's own garbage as well as that of others' found along the way. They talked about what would happen if the group did not do so, typically referring to how the landscape would turn into a garbage pile if everyone visiting left even only one or two things behind.

The observation of guides addressing concepts of sustainability mainly through their focus on “leave no trace” and “take only pictures, leave nothing but footprints” is corroborated by their reflections in the post-trip interviews. Although the degree to which they themselves claim to focus on leave no trace varies between the guides, it comes across as their main way of addressing concepts of sustainability in their guiding practices. For some of the guides, the first briefing is the only time that they mention “leave no trace” and they do not enforce it rigorously during the trip.

When tourists were asked if and how they felt that their guides highlighted concepts of sustainability or environmental issues, those who could be specific mentioned the way guides emphasized “leave no trace” throughout the trip, as well as the introduction and use of “doggie-bags.” For both international and Norwegian tourists, concepts of sustainability became a matter of “leave no trace,” an experience in nature that is run in a way so that future generations can have the same experience in the same environment, and recycling.

### Modeling Sustainability Performance

Some of the guides emphasized that they deliberately try to “model environmental behaviour”; that is, during briefings they would stress the need to make sure not to leave any trash behind, but they would not mention the possibility of tourists picking up trash found along the way. Instead, they would do that themselves and through that, model a behavior that made picking up trash and cleaning up nature “second nature,” something one just did. A few of the tourists mentioned how they felt that the guides “modeled behavior” through staying on the path, not littering, and picking up other people's litter along the path.

All of the guides believed they could, to some extent, influence tourists' environmental attitudes and behaviors. They acknowledged that their influence might not be lasting nor necessarily very profound, but nevertheless positive. Four of the guides believed their influence stems from modeling behavior and also from “modelling appreciation” for nature, such as by enthusiastically emphasizing the beauty of the surroundings, the taste of blueberries picked, the smell of the mountain moss. One guide, however, believed that taking part in nature-based adventure tourism trips itself is sufficient for strengthening tourists' sustainability and environmentally-friendly behaviors and attitudes. This guide favored “seeing and being” in nature as the primary influence, not what guides do or don't do. In this guide's view, “seeing and being” gives tourists a deeper appreciation of the natural world which, in turn, could lead them practice sustainability more in their everyday lives.

### Thinking About Sustainability

When asked whether they felt sustainability and related themes were topics for discussion during the trip, most of the tourists gave ambiguous responses. While most did not discuss sustainability, many of them (particularly internationals) felt that sustainability was omnipresent on the trip, mainly in the form of “leave no trace.” At the same time, most of the tourists claimed to be environmentally conscious and that issues related to sustainability and environmental topics both concerned and, in many cases, affected them in their daily life. When asked to exemplify, most of them mentioned a general concern about issues such as over-use of landscape and that they “do their part – I/we recycle.”

The international tourists were more specific about how their understanding of sustainability influenced their everyday life (e.g., they engaged in the public discourse on sustainability in their local communities) and how it influenced them as tourists (e.g., by paying a carbon tax for air travel).

### Supporting Others' Sustainability Performances

Not leaving any trash behind had some consequences for the guides. More than once on the skiing trips one or more of the tourists lost paper-wrappings in the wind. Each time, someone in the group would yell and make everyone aware of what was happening, and a guide would sprint off to catch the trash. When successful they were greeted with applause and loud compliments by some tourists. Other tourists' gestures – shrugged shoulders and facial experiences – and muttering indicated that they thought those applauding was making a big deal out of something unimportant.

### Minimizing Environmental Degradation

During late summer and fall hiking trips, the guides emphasized the need to stay ‘on-trail’. They explained that if everyone walked outside the path they would contribute to erosion and possibly to establishing new, unnecessary paths that contribute to environmental degradation.

### Reflection on Human/Nature

At one point during a trip, while on a scenic saddle overlooking a large, deserted beach with cliff-faces towering several hundred metres into the air, one of the guides instructed the tourists to sit down in solitude and take in the vista, the landscape, the smells, and the sounds. He encouraged them to do so for ~5 min without engaging with the others. All of the tourists except one complied with guide's instructions; one person walked around taking photos instead. The guide later said that he believed facilitating “sit-downs” and solitude reflections potentially could enhance the nature-experience for the participants and that taking in the beauty of the scenery could have a positive impact in terms of valuing the preciousness of the landscape and consequently its need to be preserved. He linked this “sit-down” with a talk he had planned later that same day addressing the issue of plastic pollution in the ocean and in general. This was the only time during the five different fieldtrips that the field researcher observed any of the guides deliberately facilitating such activities. After the “sit-down,” the guide invited the tourists to find their own path down to the beach below and to meet up by the shore at a given time for lunch. This gave the tourists opportunities to connect with nature on their own terms.

### Choosing Tour Operator

Most of the tourists acknowledged that sustainability is not of major importance when they choose a tour operator and destination. It was important for a few of the international tourists. For these people, sustainability was understood broadly, encompassing environmental, social and economic aspects.

### Learning About Nature and Culture

Compared to the Norwegian tourists, the international tourists were keen to learn as much as they could about the country and landscape. These tourists depend on the guides' local knowledge in order to get the experience they expect. The guides notice this difference between types of tourists. One informant, an apprentice guide fresh from training, observed that most international tourists are about “seeing it,” while some are also into “being there” which he thought was a deeper and better way of experiencing a landscape or destination. By contrast, this guide felt that Norwegian tourists on the same trips are more about “being” on the trip, or in a Norwegian sensibility, “doing” friluftsliv: doing, seeing and experiencing things together with friends.

The more experienced guides echoed this view and added that as guides they have to deal with the two groups differently. Some of the guides were explicit that it was much “easier” to work with international tourists because they are generally more enthusiastic about the planned trip and related activities, including learning about new culture, nature, landscape, and traditions. The guides felt international tourists generally asked more questions. However, the guides offered relatively few opportunities for tourists to learn about the local environment and culture. There was occasional storytelling by the guides, but storying the landscape in terms of history, geography, geology, biology, or culture was not a central part of the guides performances. Rather, their focus was on gazing upon the landscape and traveling through it for enjoyment.

What became evident in interviews with the tourists was that their acceptance of the guides' focus varied greatly among them. Some would not mind more emphasis on history, culture and landscape and some were quite happy with the status quo. A third group wanted as little input from the guides as possible, because they preferred to see the landscape for themselves and experience the trip as described by the tour company.

### Responding to Global Issues

As stated above, when sustainability was brought up in discussion, it was mostly by one or other of the international tourists. Often, it would be as a specific question of the guide or researcher, such as “how is Norway affected by climate change?,” or “do Norwegians think about their carbon-footprint?”

Global issues relating to climate change concerned several of the international tourists who acknowledged the dilemma of wanting to travel to pristine destinations while knowing that doing so would leave a significant carbon footprint. Some of these people stated that they had recently put planned travel on hold because they did not feel comfortable about the carbon-footprint required to get to the desired destination. In a similar way, some of the international tourists expressed concern about travel that they thought would contribute to (over)populating the chosen destinations; this concern had led, in a few cases, to decisions to drop their plans all together due to the number of other tourists expected to be at the same destination.

The Norwegian tourists, too, were conscious of the carbon-footprint of flying to destinations, but as a group they were less clear about how they understood sustainability and most of them acknowledged that it was not a major factor in their decisions and practices.

### Not Sustainability

While we did find performances of sustainability in our data, sustainability was not a major focus for the tourists. What does appear to be in the foreground for both the international and the Norwegian tourists are the experiences they are taking part in at the moment, the experiences that are to come in the near future (later that day, or the next day), and how these experiences are felt. After a long day out hiking, biking or skiing, the tourists' focus was on re-living the day's experiences and sharing feelings and thoughts about them. In these discussions, only sometimes initiated and led by the guides, the vantagepoint of experience was “the self.”

We found the same low attention to sustainability among the guides. In the main, they do not emphasize it as a topic of interest or concern in their briefings, nor during the more leisurely talks and discussions with their tourists. Overall, the guides' main focus seemed to be on practical information regarding the immediate needs for the day's journey. In particular, when briefing and talking with international tourists, the guides focused on providing detailed information about technicalities of the forthcoming activities, such as the quality of the path (gravel, loose rock etc.), altitude gain/loss, distance to be covered, safety concerns and how to deal with them, expected pace, when and where to eat the bagged lunch, how to dress, what to have in the backpack in terms of spare clothing and other accessories, what they could expect to see during the day, and why this experience would be worthwhile. When engaging with Nordic tourists, the guides provided the same type of information but with less detail, as if they expected the Nordic tourists to be more familiar with the weather, equipment and environment.

Although the five different trips took place in different landscapes, at different times of the year, using different adventure activities, the way the tour days were organized was very similar. Each day began with a shared breakfast usually followed by a short and practically-oriented briefing about what was ahead, then some time to pack personal gear, and meet at a designated location at about 9 a.m. The activity of the day usually lasted around 8–10 hours and ended with supper at around 7 p.m. Each day's journey had a similar pattern: hiking, biking, or skiing for 50 minutes, usually in single file, before a 10-minutes break. This routine would continue throughout the day, until the group reached the planned destination, and it created a conflict for the guides. Addressing the group as a whole while hiking, biking, or skiing was a demanding and difficult exercise for the guides because they were left with 10 in every 50 minutes as their “window of operation.” In this time, they had to monitor the group and individual well-being, attend to issues such as broken equipment, adjusting backpacks or skis, taping up blisters, and make sure that they engaged in at least one conversation with each participant each day. Several of the guides emphasized in their interview that they were reluctant to overtly interrupt the breaks with information about landscape, culture, history, or sustainability, because they wanted to allow individual participants to make use of the break as each saw fit.

Further, the guides felt challenged to tread a fine line between enhancing the tourist experience while at the same time not appearing to “have an agenda” or creating a “situation” that the tourists had not signed up and paid for. Many of the respondents also said that an outspoken sustainability and environmental focus from the guides could easily be interpreted as moralizing, which they were neither interested in nor positive toward. Several of the guides stated in various ways, both during the trip and in post-trip interviews, that their primary task was to make sure the tourists had a good time on their vacation. In fact, the guides stressed the view that the tourists were on vacation, implying that being on vacation imposed some guidelines in terms of a guide's behavior.

## Discussion

We set out to investigate if and how tourists and guides understand, operationalize, practice, and embody deeper nature-relatedness, active environmental and cultural protection, and relationship to the global collective. We found that performances of sustainability are not a major component of guides' and tourists' performances while on tour. Of the sustainability performances that we did find, the guides and tourists practiced and embodied nature-relatedness at both shallow (everyone noticing nature) and deeper (some tourists seeking isolation and reflecting on human/nature) levels. They expressed a limited range of environmental protection actions (reducing pollution by picking up garbage, minimizing environmental degradation by staying on tracks) and international tourists expressed interest in local culture which is one aspect of motivation for cultural protection (Calver and Page, 2013; Richards, 2018). Further, we found that international tourists and, to a lesser extent Norwegian tourist, expressed interest in global issues (mainly carbon footprint), which is arguably a signal of positive relationship to the global collective. In addition to these types of sustainability, a few tourists chose the tour operator with sustainability in mind, however our data does not indicate which aspects of sustainability informed those choices. Finally, tourists and guides expressed an over-arching thoughtfulness on sustainability: they all thought about it, some guides modeled it, and tourists supported the guides' modeling. However, these mainly cognitive actions did not apparently lead to additional expressions of sustainability by the tourists.

We understand the variability in expressions of sustainability through a Goffmanian lens of four **distinct clusters of frames:** one cluster is made up from the Norwegian tourists; another from the international tourists; a third from most of the guides; and the fourth from one particular guide. Goffman's ideas of “going about” normal life and “being alert” to threats and changes are useful for describing these frames. In the Norwegian tourist frame, going about nature-based adventure tourism means performing friluftsliv while being guided, connecting with nature individually, and not being disturbed (threatened) by issues beyond the immediate enjoyment of activity and environment. By contrast, the international tourist frame seeks out the challenge of difference (e.g., curiosity about Norwegian culture and history) and environmental threat (e.g., climate change) while also enjoying the immediate activity and environment. Most of the guides shared a frame that fits/matches that of the Norwegian tourists: a “normal” guide allows tourists to go about their tourism without being alarmed by the intrusion of overt sustainability performances by the guides. The fourth evident frame was that of a single guide who considered nature-based adventure tourism to normally involve challenging tourists' perceptions of sustainability. Clearly, these four frames are not all, always, compatible, which suggests that the guides and tourists reached a common expressive order for the trips. This consensus revolved around enjoyment, as we now discuss.

### Sustainability Performances vs. Enjoyment

Through both interviews and comments made during the different tours, it is evident that a primary aspect of the guides' frame is prioritizing tourist enjoyment within the scope of the planned trip. Enjoyment is central to the interaction order of these situations. The guides express a high degree of awareness of the fact that the tourists have paid to get a certain product. The product is defined in terms of sites to see, places to visit, adventure activities to do, and more generally when, what and how the different aspects of the trip are supposed to take place. These details are stated in the written “contract” - detailed information about the content of the given tour - on the tour operator's website that tourists access before the trip. This “contract,” then, is the tour company's frame for the particular trip: it provides the “social information” (Goffman, 1967) that helps tourists and guides to understand “what sort of situation [this is]” and, consequently, what sort of performances are expected of them. The “contract” tells tourists what they can expect to happen and to experience. It tells guides what they have to deliver. Through both interviews and field conversations it is clear that the guides see their work as contractual and that they feel obliged to deliver a “product” as close to the “contract” as possible. In Goffman's (1959) terms, they conform to the “interaction order” and in doing so they prioritize enjoyment over sustainability. Their emphasis is on facilitating a relaxed, friendly and positive social atmosphere within the group and making sure that the tourists have a good time and enjoy themselves. It is only if and when tourists express enjoyment of deeper sustainability that the guides respond. Thus, it is the tourists who must first challenge the interaction order; the guides follow tourists in opening up for deeper sustainability. Larsen and Meged (2013, p. 101) argue that it is tourist's “participatory and attentive tactics” that turn guided tours into co-created performances. Larsen and Meged (2013, p. 101) also found that “guides rely on the energy from interactions and participants which is why the guiding is equally affected when the tourists log off.” A possible explanation to why the “interaction order” seems to stay fairly fixed on enjoyment in the tours we observed, could be that the guides are sensitive to tourist “logging off” if addressing or emphasizing deeper sustainability performances when not initiated by the tourists themselves.

As noted in the results, we did identify one performance by one guide that might have challenged the tourist's perceptions of sustainability and thus also the “interaction order.” This was the invitation to sit and reflect, then to find one's own way to the beach and take some time there. As this episode took place on a trip with international tourists, it is pertinent to ask whether guides use different frames depending on what type of tourist groups they guide, whether international tourists tend to challenge the interaction order more, and if so, how these challenges are resolved. These questions will be the focus of a future article.

A primary focus on enjoyment, however, does not preclude other foci, less central to the frame. For some guides, a focus on sustainability was possible as long as it didn't interfere with enjoyment. In the next section we discuss susceptibilities that produce potential for more, or deeper, sustainability.

### Susceptibility to Sustainability

Some of the guides expressed that their understanding (or frame) of the trip and, therefore, their potential scope of action, differed based on the type and length of the trip they were guiding. One guide mentioned that trips longer than 2 weeks provided more opportunities to address a broader range of topics because there is more time to interact with individual tourists. While no such trips were the subject of this study, the guide's comments throw light on a way that guides can manage social interaction for particular effects. This guide explained that:

“. in the end you deliver a product that someone has paid for. so you need to know your group … Some are very susceptible for discussions and new ways of thinking, others find it annoying … So I don't push [sustainability issues/practices] so much, but do more sort of systematic brainwashing [laughs out loud]. because you spend quite a lot of time with the tourists, and then you can lead them, in the direction that you would like to see them end up … And that is not something you do the first day. It takes time.”

Following Goffman (1974), one explanation for this guide's comments is that guides can have multiple backstage topics that they intend to emphasize throughout the trip and which, through planned performances, can gradually become front-staged, possibly without the tourists noticing the shift. In other words, sustainability could be an aspect of the guide's frame for the trip from the outset, but he or she keeps it “backstage” (Goffman, 1959) until they feel that the tourists are ready (“susceptible”) for it. By back-staging sustainability, this guide managed the impression of himself so that his “front-stage” (Goffman, 1959) performance matched his perception of tourist interest in sustainability, and this saved the tourists' “face” rather than creating an uncomfortable or embarrassing situation. However, this explanation fails to address how tourists become more interested in the guide's prepared topics. If this static view of frames is adopted, the question of how tourist frames can be made more susceptible to sustainability remains open. It also calls into question how the guides ascertain tourist susceptibility.

Taking into consideration the guides' educational backgrounds, it could be that this guide did actually have a deliberate educational program in mind in his “backstaging-to-frontstaging” of sustainability. In fact, five out of the six guides in this study have attended nature guide-related educational programs at university level in Norway. Andersen and Rolland (2018) argue that nature guides educated in friluftsliv (as is the norm in nature-based higher education courses in Norway) can “add value by enhancing participant's experiences and adding more learning to the experience. The learning relates to skills and techniques … and connecting the participants more closely with nature” (p. 1). However, Weiler and Kim (2011) argue that because tour guides, in general, have limited exposure to or experience with “theory, tools, and techniques for optimizing the visitor experience and visitor-environment interaction within a sustainability framework” they might not be “fully realizing their potential to communicate and role-model sustainability in their tour content and practice” (p. 113). In our view, there is merit in asking if the guide education programs in Norway do provide the necessary “theory, tools, and techniques” required for framing sustainability in their professional roles.

Goffman (1967) highlights the importance of the communication process in “the nature of the ritual order” (p. 42). It is the communication process that takes place between the guides and the tourists that is important for explaining “what it is that's going on” and according to Goffman this is largely due to feelings. Feelings are “vulnerable not to facts and things but to communications” and “[c]ommunications … can be by-passed, withdrawn from, disbelieved, conveniently misunderstood, and tactfully conveyed” (Goffman, 1967, p. 43). The trust that the guides build through their individual way of communicating with the tourists creates the potential scope for action to discuss sustainability. If the communication between the parties involved is not open and trusting, the possibility of maintaining an expressive order is made more difficult. The longer a trip lasts, the better everyone gets to know each other, which then gives room to expand the repertoire of what it is acceptable to talk about.

In our study, most tourists framed the trips in non-sustainability ways, as did most guides. However, occasionally guides were prompted by tourists to focus on a deeper sustainability at least with regard to learning about environment and culture, or when they received positive feedback from the tourists such as when they were applauded for retrieving trash. At those times, the guides at least attempted to respond in a deeper sustainability way themselves. Conversely, when the tourists were invited to deepen their relationship with nature by taking a “sit-down and reflect,” their framing of the trip might have shifted or widened to encompass a (slightly) deeper focus on sustainability. None of them reported that it did, however. One possible reason could be that, as all the tourists on this particular trip were non-Nordic and well-experienced in nature-based adventure tourism, they might have already reached a deep-enough level of sustainability practice that such reflection is normal and not note-worthy. If so, this particular trip could be considered similar to many eco-tourism trips which have been challenged for “preaching to the converted” rather than increasing the public's exposure to deeper sustainability experiences (Beaumont, 1991).

Several of the guides found Norwegian tourists in Norway to be less interested in learning from the tour and more critical toward the guide. This difference apparently has an effect on both how the guides perform their guiding, and the guides scope of action. With Norwegian tourists, the guides often felt the need to prove their competence while at the same time sensing that many of the Norwegian tourists felt they did not actually need a guide. Also, working with the less enthusiastic (Norwegian) tourists affected how the guides behaved and their guiding style. It seems that working with a group with the same cultural background poses some challenges for the guides in terms of what to focus on in their guiding practice. This possibility is worth further investigation for its impact on the sustainability potential of domestic nature-based adventure tourism.

### The Complication of Tour-Logistics

We turn now to consider guides' framing and its relationship to the tour company. Weiler and Black's (2015) observation that tour companies can leave guides little power to perform sustainability on any given tour is pertinent to this discussion. In our study, the way the tours were organized left little time for performing sustainability that was not already framed by company.

The tour logistics emphasized: (1) the adventure activity itself (hiking, biking, skiing), (2) gazing (Urry, 1990) upon the landscape, and (3) journeying through the landscape. The tour logistics, in our interpretation, are framed as getting the tourists from point A to point B. Performing nature-based adventure tourism seems to mean giving the tourists what they had paid for. The different sustainability performances we did observe mostly took place during the adventure activities, not as planned nor *pre*formed performances linked to the company's programme, but rather as spontaneous performances that took place *in situ*. Only on a few occasions did we observe the guides choosing to facilitate sustainability. In our study, then, sustainability was inspired mainly by the responses of tourists and guides to their immediate experiences of the adventure activities, within particular settings. Sustainability actions and practices were not emphasized strongly in the orchestration of tour logistics.

The tour company involved in this study did not “frame” sustainability as part of the experience of the trips. That we found sustainability performances in our data suggests that tourists are “ready” for “light” sustainability at least. Arguably, this company and others would not damage their reputations by promoting the level of sustainability that tourists will happily accept. By framing sustainability into the experiences, tour companies would also be opening up possibilities for guides to frame their work for deeper sustainability.

## Conclusions

This study has shown that sustainability, as we understand it, did occur at the micro-level of the nature-based adventure tourism experiences we studied in Norway, albeit as a minor theme in guides' and tourists' framing of trips. The sustainability performances we found mainly sprang from spontaneous responses by tourists and guides to experiences of adventure activities in particular natural settings. We have shown that sustainability performances can be ambiguous, complex and contingent upon the interplay of guides' and tourists' frames.

Nature-based adventure tourism companies appear to be key agents in the framing of trips by both guides and tourists. There appears to be potential for deeper sustainability to be expressed on guided trips if companies allow it. The implications for promotional messaging and expectation-setting through pre-trip interactions with tourists are worthy of further investigation. Similarly, there are implications for guide training and for the knowledge and skills demanded by nature-based adventure tourism companies of their guides.

Deeper sustainability might be found more readily in situations of “foreignness” or difference, such as among international tourists. This possibility needs further exploration. If susceptibility to sustainability is greater in “foreign” contexts, how can the tourism industry respond? This question seems especially pertinent in the current relatively closed global context and in the prospect of international travel in the foreseeable future being limited by cost and pandemic controls.

While not generalizable to other settings, our findings demonstrate that sustainability in tourism can be empirically studied by taking a performative ethnographic approach in field work. Further studies in a wider variety of settings, and especially longer trips, could potentially tease out some of the ambiguities and complexities we have noted. Study designs that access tour operators', guides' and tourists' perceptions of one another's frames would shed additional light on the ways in which these actors influence one another's sustainability understandings and actions. Finally, studies that access guides' and tourists' longer-term reflections on trips might also bring to light important aspects of trip dynamics on sustainability in their everyday lives.

This paper presents a study of how sustainability is operationalized in a nature-based adventure tourism setting. The study is novel in its method, empirical data, and Norwegian setting. The results are relevant to the national as well as the international tourism industry.

## Data Availability Statement

The raw data supporting the conclusions of this article will be made available by the authors, without undue reservation.

## Author Contributions

ARo, PL, and ARa contributed to the research idea and design of the study. ARo conducted the fieldwork and data analysis under supervision of the other authors. ARo wrote the first draft of the manuscript. All authors contributed to manuscript revision and approved the submitted version.

## Conflict of Interest

The authors declare that the research was conducted in the absence of any commercial or financial relationships that could be construed as a potential conflict of interest.
